# Management of Pancreatectomy for Pancreatic Cancer in a Patient With Annular Portal Pancreas: A Case Report

**DOI:** 10.7759/cureus.85950

**Published:** 2025-06-13

**Authors:** Hiroyuki Hakoda, Koichiro Kawasaki, Kiyohiko Omichi, Keiichi Nasu, Kentaro Inada, Michiro Takahashi

**Affiliations:** 1 Department of Surgery, Tokyo Metropolitan Bokutoh Hospital, Tokyo, JPN

**Keywords:** pancreatic abnormality, pancreatic fistula, pancreatic malformation, rare variation, surgical complication

## Abstract

Portal annular pancreas (PAP) is one of the rare pancreatic anomalies in which the pancreatic parenchyma surrounds the portal vein (PV) or superior mesenteric vein (SMV), accounting for only around a few proportions of all patients. PAP is thought to be associated with the high risk of postoperative pancreatic fistula (POPF) after pancreatectomy. We describe our experience of a case with PAP and review the literature on pancreatectomy in patients with PAP. A 72-year-old male presented to our department with a pancreatic body mass with a history of previous abdominal surgeries, who underwent distal pancreatectomy (DP) with lymphadenectomy following neoadjuvant chemotherapy with gemcitabine plus nab-paclitaxel. PAP was identified during surgery, which was not found in computed tomography scans and other modalities in the previous examinations. The annular pancreas was resected using tri-staplers with polyglycolic acid (PGA) sheets. His postoperative course was uneventful without POPF, and he was discharged on postoperative day 11. In conclusion, when PAP is suspected in patients with pancreatic cancer, understanding the accurate anatomy of the pancreas is essential to determine the surgical technique and a suitable choice of device for the transection of pancreatic parenchyma for reducing POPF.

## Introduction

Portal annular pancreas (PAP) is a rare congenital anomaly in which the portal vein (PV) or superior mesenteric vein (SMV) is encircled by pancreatic parenchyma [1,2]. This condition is thought to arise from pancreatic maldevelopment due to abnormal fusion of the dorsal pancreatic bud with the ventral pancreas [3-5]. According to previous reports, the incidence rate of PAP ranges from 0.8% to 3.5% among all patients [3,6-8]. Most cases of PAP are asymptomatic and incidentally diagnosed using computed tomography or during surgery [9]. Preoperative diagnosis is often difficult because PAP remains unrecognized, although the entity of PAP is well described in the literature [10]. Several classifications of PAP have been proposed based on the anatomy of the main pancreatic duct (MPD) [1,10]. Resection in patients with PAP has been associated with a potentially higher risk of postoperative pancreatic fistula (POPF) [3,8,11,12]. Given that pancreatic anomalies are a known risk factor of POPF, it is essential for surgeons to carefully determine the resection level of the pancreas and select appropriate procedural techniques for pancreatectomy, such as using an autosuture device or crushing method. Herein, we report a case of PAP in a patient with pancreatic cancer who underwent distal pancreatectomy (DP) without POPF and provide a review of the literature.

## Case presentation

**Figure 1:**
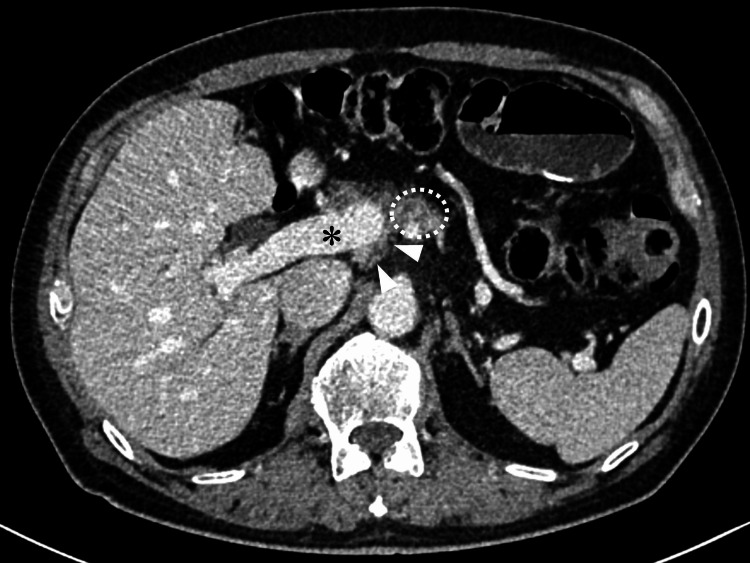
The contrast-enhanced computed tomography image before neoadjuvant chemotherapy Arrowheads indicate the dorsal pancreatic parenchyma; the asterisk indicates the portal vein; dotted lines outline the pancreatic tumor.

**Figure 2:**
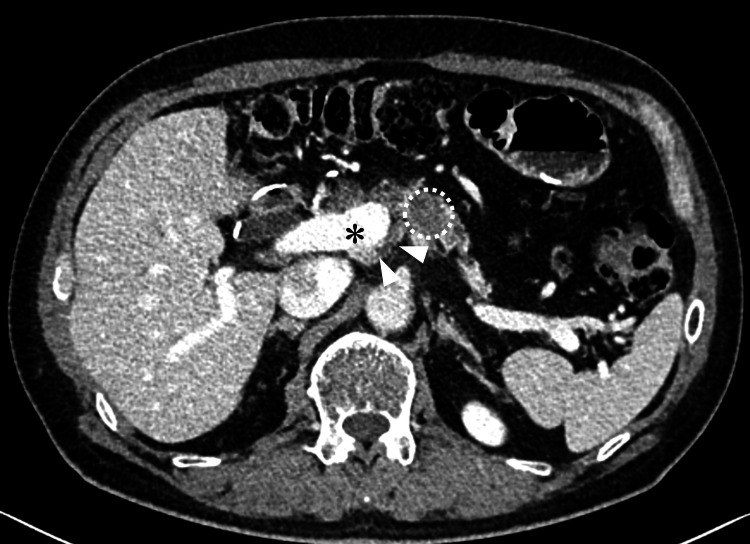
The contrast-enhanced computed tomography image after neoadjuvant chemotherapy Arrowheads indicate the dorsal pancreatic parenchyma; the asterisk indicates the portal vein; dotted lines outline the pancreatic tumor.

A 72-year-old male was referred to our hospital with a pancreatic body mass suspected to be pancreatic cancer. His medical history included laparoscopic distal gastrectomy for gastric cancer 14 years earlier and laparoscopic anterior resection for rectal cancer 10 years earlier. The tumor location and prior gastrectomy made it impossible to perform endoscopic ultrasound-guided fine-needle aspiration due to the tumor’s distance from the stomach. The periportal pancreatic parenchyma could not be detected in endoscopic ultrasonography. Therefore, the diagnosis of pancreatic cancer was made based on imaging. The tumor was considered biologically borderline resectable due to an elevated carbohydrate antigen 19-9 level (maximum: 1,779.2 U/mL) [13]. A multidisciplinary team recommended neoadjuvant chemotherapy with gemcitabine and nab-paclitaxel. CT before surgery showed soft tissue on the dorsal side of the PV near the pancreatic tumor (Figures 1, 2), which was initially interpreted as tumor-associated inflammation.

**Figure 3:**
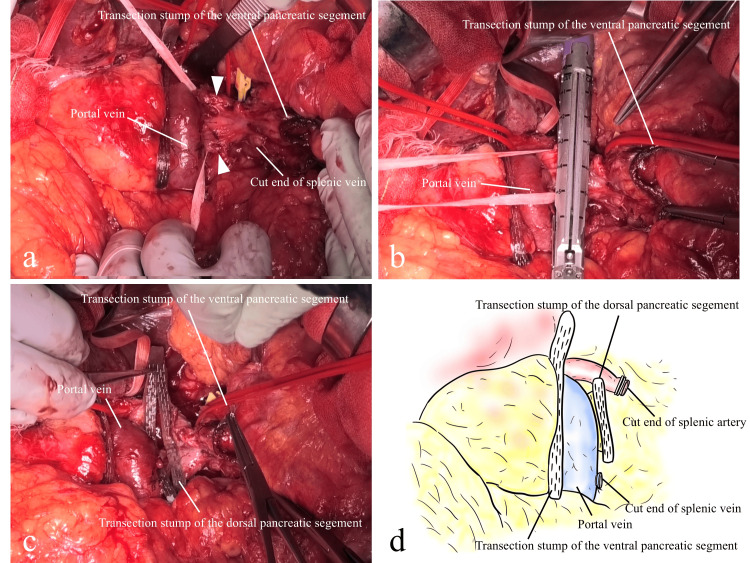
Intraoperative findings of portal annular pancreas (a) Arrowheads indicate the dorsal pancreatic parenchyma running posterior to the portal vein. The yellow clip is clamping the splenic artery. (b) Transection of the dorsal pancreatic parenchyma is shown. The findings of the transection of the ventral pancreatic parenchyma with a tri-stapler. (c) The findings after transection of both ventral and dorsal pancreatic parenchyma with tri-staplers. (d) Schematic illustration of the intraoperative findings after transection of both ventral and dorsal pancreatic parenchyma. Transection was performed using tri-staplers with polyglycolic acid sheets.

After receiving neoadjuvant chemotherapy with two cycles of gemcitabine plus nab-paclitaxel, the patient underwent DP with lymphadenectomy. The operative time was 348 min, and the estimated blood loss was 316 mL. Intraoperatively, the annular pancreas was identified and resected using a tri-stapler with polyglycolic acid (PGA) sheets [Signia™ Reinforced Reload with Tri-Staple technology, a purple cartridge (Medtronic, Minneapolis, MN, USA)] in two separate transections. Initially, the pancreas encircling the SMV was divided using the tri-stapler with PGA sheets (a purple cartridge). Pancreatic tissue was found dorsal to the PV (Figure 3a), and intraoperative ultrasonography (IOUS) confirmed the absence of MPD in this region. Based on these findings, a diagnosis of type IIIa PAP was made. The dorsal pancreatic parenchyma was mobilized from the superior mesenteric artery (SMA) nerve plexus, safely taped, and then transected using the tri-stapler with PGA sheets (a purple cartridge) (Figures 3b-3d). DP was performed safely, with no damage to the remnant pancreas. Despite a previous gastrectomy, the remnant stomach was preserved, and blood flow was confirmed by indocyanine green fluorescence imaging. The patient had an uneventful postoperative course and was discharged on postoperative day 11 without POPF. Histopathology confirmed T3N1M0 stage IIB pancreatic cancer (Union for International Cancer Control, 8th edition) with negative margins. He has been subject to follow-up as an outpatient and has remained recurrence-free following adjuvant chemotherapy with S-1.

## Discussion

This case describes a patient with PAP who was intraoperatively diagnosed and successfully underwent DP for pancreatic cancer without developing POPF. Key aspects of this case include accurate intraoperative identification and classification of PAP as type IIIa, in which the uncinate process fuses with the pancreatic body and surrounds the PV cranial to the splenic vein, and safe resection using a careful two-cut stapling approach. The patient’s outcome was favorable, with no postoperative complications.

PAP is a congenital anomaly caused by abnormal fusion of pancreatic buds during development [1,4,7,10,14]. In contrast to annular pancreas encircling the duodenum, which can cause obstruction and symptoms, PAP is usually asymptomatic [4,7,11,15]. Therefore, they are often identified incidentally during imaging or surgery [9]. A commonly used classification categorizes PAP into supra-splenic, infra-splenic, and mixed types, depending on the relationship between the encircling pancreatic tissue and splenic vein [1]. Among these, the most common type is IIIa [16], as observed in the current case.

Although PAP is a known anomaly, preoperative diagnosis remains difficult, even with CT or magnetic resonance imaging (MRI) [10,14,17]. Studies report that up to 50% of PAP cases are diagnosed incidentally during surgery [7]. The reported incidence of PAP ranges from 0.8% to 3.5% [3,6-8]. It may present as a retroportal mass, which can complicate its recognition on imaging and make preoperative diagnosis difficult. Although some studies have shown that advancements in imaging modalities have improved the ability to identify PAP before surgery [8,10,18], diagnostic accuracy remains limited, with one study reporting a false-negative rate as high as 52.9% [3]. Magnetic resonance cholangiopancreatography has been suggested as a useful tool for identifying PAP and delineating the anatomy of the MPD prior to surgery [10,16,17]. However, in the present case, preoperative identification of PAP using conventional imaging techniques, including CT and MRI, was not feasible.

When PAP is encountered during DP, special attention is required in managing the dorsal pancreatic parenchyma, as anatomical variations may influence both surgical decision-making and outcomes. Two main concerns arise in such cases: determining the appropriate transection level and selecting the surgical approach. Prior studies have described two possible transection strategies: one involves resection on the distal left side of the PV/SMV (the one-cut margin method), and the other entails separate transection of both ventral and dorsal pancreatic segments (the two-cut margin method) [3,19]. However, consensus on the most appropriate technique remains lacking. Surgical approaches for managing PAP in DP have varied across previous reports [4,5,9,15,18,19]. Although some authors have advocated for the one-cut margin method due to its lower reported incidence of POPF in certain cases, others have argued that this technique may increase risk due to a larger resection surface and potential for unintended injury to pancreatic tissue [8,19]. Conversely, the feasibility and safety of the two-cut margin method have also been demonstrated in other reports [8,17]. This technique may be advantageous because the pancreatic parenchyma at the level of the PV/SMV tends to be thinner than in other regions, making it a suitable site for transection and potentially reducing POPF risk, provided the tumor location permits it.

Two main options exist for surgical transection: manual division with suture ligation or use of an autosuture device. In the former, precise identification and ligation of the MPD are essential to minimize the risk of POPF [8,9]. In cases of type I or II PAP, where the MPD courses dorsal to the PV/SMV, special caution is required when managing the dorsal pancreatic bud. One study reported an increased incidence of POPF when the dorsal pancreatic bud was transected without a stapling device [15]. Recently, several studies have demonstrated that using autosuture devices such as triple-row staplers can lower the incidence of POPF in pancreatectomy [15,20-23]. These results support the use of stapling devices, particularly in anatomically complex or high-risk scenarios such as PAP.

In the present case, PAP was not diagnosed before surgery. However, IOUS enabled detailed and accurate identification of the anomaly. In the present study, the two-cut margin method was selected for two main reasons: first, the tumor was located in the pancreatic body and required resection at the PV level to secure a clear margin; second, the dorsal pancreatic parenchyma was only identified after the resection of the ventral segment. The pancreatic parenchyma along the transection line was sufficiently thin to allow both ventral and dorsal portions to be safely divided using an autosuture device, without damaging the remnant pancreas.

This is a single case report supplemented by a literature review. Given that PAP is rare, large-scale studies have not been conducted. Additional research is needed to explore optimal surgical techniques for reducing POPF risk in patients with PAP.

## Conclusions

Although PAP is a well-known congenital anomaly, it remains uncommon and poses a significant risk factor for POPF. Careful anatomical assessment is crucial for reducing postoperative complications, particularly in cases involving PAP. Based on the findings in this case, the use of a tri-stapler may offer a safer option for pancreatic transection during DP in patients with PAP. As this is a single case report, further case accumulation is required to confirm these findings.
